# Supplementary material for: Analysis of intracellular and intercellular crosstalk from omics data
Source: PLoS One. 2025 Oct 22;20(10):e0334981. doi: 10.1371/journal.pone.0334981 (PMC12543113; doi:10.1371/journal.pone.0334981)
Supplement: S1 File — (PDF) [file pone.0334981.s001.pdf]

SUPPLEMENTARY INFORMATION

Analysis of intracellular and intercellular crosstalk from omics data

Alice Chiodi[1], Paride Pelucchi[1], Ettore Mosca[1*]

[1]Institute of Biomedical Technologies, National Research Council, Segrate (Milan), Italy

*Corresponding author

E-mail: ettore.mosca@itb.cnr.it (EM)

Table of contents

SUPPLEMENTARY TEXT 3

S1 Text. Definition of crosstalk and its saturation *3*

S2 Text. Statistical assessment of crosstalks *3*

S3 Text. Definition of crosstalk diversity and interaction diversity *4*

SUPPLEMENTARY FIGURES 6

S1 Fig. Gene weights, gene-gene interactions and crosstalk values of four gene sets pairs that exemplify the four combinations of evidence about the statistical significance of a crosstalk based on the two null models MA and Mu. *6*

S2 Fig. Frequency distribution of the Spearman correlation values between the two null models MA and Mu. *7*

S3 Fig. Comparison of cell clusters and cell type annotation of the 8 TNBC samples used in our proof-of-concept. *8*

SUPPLEMENTARY TABLES 9

S1 Table. Estimated probabilities of the crosstalk values reported in S1 Fig. *9*

S2 Table. Differential expression analysis results. *9*

S3 Table. Differential expression genes (DEG) mapping data. *9*

S4 Table. Intracellular crosstalk results in Cancer cells. *9*

S5 Table. Over-Representation Analysis (ORA) on cancer cells DEGs results. *9*

S6 Table. Gene classification of cancer intracellular crosstalk. *9*

S7 Table. Intercellular crosstalk results. *10*

S8 Table. Cancer-CAF intercellular crosstalk details. *10*

S9 Table. Intercellular crosstalk gene classification. *10*

S10 Table. Integrated crosstalk analysis in cancer cells, involved in communication with CAF. *10*

REFERENCES 11

Supplementary Text

S1 Text. Definition of crosstalk and its saturation

Here, we describe how we quantify a "crosstalk" between two gene sets. We consider the general scenario where input data derive from single-cell RNA-sequencing experiments, because this enables the calculation of both intra-cellular and extra-cellular crosstalks[1]. In this case we have input vectors $(\mathbf{u}_1, \dots, \mathbf{u}_\mathrm{m})$, each of which contains gene-level scores that characterize the "profile" of a cell type (or cluster), like, differential expression scores between a cell type and the others. Let us consider:

- the universe $\Sigma = \{X_i\}_{i=1}^{\mathrm{N_S}}$ of gene sets X that we want to analyze; the calculation of *intra-cellular crosstalk* requires $X \cap Y = \emptyset \;\; \forall (X, Y) \in \Sigma^2$;
- the totality of genes, which forms the set $V = \bigcup_{i=1}^{\mathrm{N_S}} X_i = \{g_i\}_{i=1}^{|\mathrm{N_G}|}$, indexed by the set $I = \{1, \dots, \mathrm{N_G}\}$;
- the undirected graph $G = (V, E)$ among the genes, where E are gene-gene interactions, and the corresponding adjacency matrix $\mathbf{A} \in \{0,1\}^{\mathrm{N_G} \times \mathrm{N_G}}$;
- the $\mathrm{N_G} \times \mathrm{m}$ weight matrix $\mathbf{U} = (\mathbf{u}_1, \dots, \mathbf{u}_\mathrm{m})$, $\mathbf{U} \in [0, 1]^{N_\mathrm{G} \times \mathrm{m}}$;
- the weight function w that defines the gene weight vector associated with the gene set X as: $\mathbf{u}_\mathrm{X} = w(X, j; \mathbf{U}) = \mathrm{diag}(\mathbf{1}_\mathrm{X})\, \mathbf{U}_{*j}$, where $\mathbf{1}_\mathrm{X}$ denotes the indicator vector of X, that is $\mathbf{1}_\mathrm{X}(i) = \begin{cases} 1, & i \in K_\mathrm{X} \\ 0, & i \notin K_\mathrm{X} \end{cases}$, and $K_\mathrm{X} \subseteq I$ is the set of indices of $g_i \in X$.

The crosstalk of any gene set pair $(X, Y) \in \Sigma^2$ is:

$$C(X, Y) = \mathbf{u}_\mathrm{X}^\mathrm{T}\, \mathbf{A}\, \mathbf{u}_\mathrm{Y} = \sum_{i=1}^{\mathrm{N_G}} \sum_{j=1}^{\mathrm{N_G}} a_{ij}\, \mathbf{u}_\mathrm{X}(i) \mathbf{u}_\mathrm{Y}(j).$$

Typically – as explained in the main text – the calculation of *intra-cellular crosstalk* is done using the same column of $\mathbf{U}$ for X and Y: $\mathbf{u}_\mathrm{X} = w(X, j)$, $\mathbf{u}_\mathrm{Y} = w(Y, j)$.

Let us consider all pairs of sets (X, Y) that establish a crosstalk $C(X, Y) > 0$. Let us define the number of links between X and Y as $L_\mathrm{XY} = \mathbf{1}_\mathrm{X}^\mathrm{T}\, \mathbf{A}\, \mathbf{1}_\mathrm{Y}$, and the number of links between X and Y affected by a molecular change as $\delta L_\mathrm{XY} = \mathbf{b}_\mathrm{X}^\mathrm{T}\, \mathbf{A}\, \mathbf{b}_\mathrm{Y}$, where $\mathbf{b}_\mathrm{X}(i) = \mathrm{sgn}\big(\mathbf{u}_\mathrm{X}(i)\big)$ and $\mathbf{b}_\mathrm{Y}(i) = \mathrm{sgn}\big(\mathbf{u}_\mathrm{Y}(i)\big)$. The saturation of the crosstalk is defined as

$$r_c = \frac{\delta L_\mathrm{XY}}{L_\mathrm{XY}}.$$

S2 Text. Statistical assessment of crosstalks

As stated in the main text, to statistically benchmark the magnitude of an observed crosstalk value c we have to consider that it might depend on various features (e.g., gene set size, distribution of gene weights and gene degree). We focused on two null models, namely $M_\mathrm{A}$and M_u, in both of which we *preserve* gene set size, degree sequence, and the

[1] Input gene vector(s) can represent any gene-related quantities, as explained in the main text.

association between gene weight and gene degree (within the same bin over the degree sequence), and randomize, respectively, gene-gene interactions and gene weights. The null model M_{A} is designed to test the dependence of c from the network proximity of X and Y, and allows us to estimate the probability of observing a value equal or greater than c, using a set of permutations $\widehat{\mathbf{A}} = \mathbf{PAP}$ where $\mathbf{P}$ is a (gene-degree preserving) permutation matrix:

$$\rho_{\mathrm{A}} = \Pr\big(C\big(X, Y; \widehat{\mathbf{A}}, \mathbf{u}_{\mathrm{X}}, \mathbf{u}_{\mathrm{Y}}\big) \geq c\big).$$

The null model M_{u} is meant to test the dependence of c from gene weights and allows us to estimate the probability of observing a value equal or greater than c using permutations $\widehat{\mathbf{U}} = \mathbf{PU}$:

$$\rho_{\mathrm{u}} = \Pr\big((X, Y; \mathbf{A}, \widehat{\mathbf{u}}_{\mathrm{X}}, \widehat{\mathbf{u}}_{\mathrm{Y}}) \geq c\big),$$

where $\widehat{\mathbf{u}}_{\mathrm{X}}$ and $\widehat{\mathbf{u}}_{\mathrm{Y}}$ are derived from $\widehat{\mathbf{U}}$ by means of the weight function w.

Then, we combine the two probabilities ρ_{A} and ρ_{u} into the probability of observing a product $\hat{\rho}$ as small as the one observed, which is [1,2]:

$$p = \Pr(\hat{\rho} \leq \rho_{\mathrm{A}}\rho_{\mathrm{u}}) = \rho_{\mathrm{A}}\rho_{\mathrm{u}} + \int_{\rho_{\mathrm{A}}\rho_{\mathrm{u}}}^{1} \frac{\rho_{\mathrm{A}}\rho_{\mathrm{u}}}{\rho_{\mathrm{A}}} \mathrm{d}\rho_{\mathrm{A}} = \rho_{\mathrm{A}}\rho_{\mathrm{u}} - \rho_{\mathrm{A}}\rho_{\mathrm{u}} \ln(\rho_{\mathrm{A}}\rho_{\mathrm{u}}).$$

Note that p is equal [1] to the probability estimated by the so-called Fisher's combined probability test [2].

Lastly, we define a summary score for ranking, combining effect size $C(X, Y)$ and its estimated probability p:

$$s(X, Y) = -\,C(X, Y)\log_{10}(p).$$

S3 Text. Definition of crosstalk diversity and interaction diversity

Let us consider all pairs of sets (X, Y) that establish a crosstalk $C(X, Y) > 0$, a subset of which is considered significant $s(X, Y) > \alpha$, given a chosen threshold α. The list of significant, or altered, crosstalks is then used to score the genes g_i that mediate such interactions. To this aim, we introduce the gene-level quantities *crosstalk diversity* and *interaction diversity*. Define *the crosstalk diversity* of g_i as the number $\delta d_{\mathrm{X}}(i)$ of gene sets Y such that:

- Y is involved in an *altered* crosstalk with a set X that contains g_i;
- Y contains a gene g_j that mediates the crosstalk interacting with g_i.

Formally:

$$\delta d_{\mathrm{X}}(i) = \big|\big\{Y \mid g_i \in X, g_j \in Y, a_{ij}\,\mathbf{u}_{\mathrm{X}}(i)\,\mathbf{u}_{\mathrm{Y}}(j) > 0, s(X, Y) > \alpha\big\}\big|.$$

We normalize this quantity considering the gene sets Y, such that:

- Y is involved in a crosstalk with a set X that contains g_i;
- Y contains a gene g_j that interacts with g_i.

That is:

$$d_{\mathrm{X}}(i) = \big|\big\{Y \mid g_i \in X, g_j \in Y, a_{ij} = 1, C(X, Y) > 0\big\}\big|.$$

The ratio of the two numbers above is the *saturation* of the crosstalk diversity of g_i:

$$r_{\mathrm{X}}(i) = \frac{\delta d_{\mathrm{X}}(i)}{d_{\mathrm{X}}(i)}.$$

Define *interaction diversity* of g_i as the number $\delta d_{\mathrm{A}}(i)$ of genes g_j, such that:

- g_j belongs to Y, a set that establishes an *altered* crosstalk with X, which contains g_i;
- g_j interacts with g_i.

Formally:

$$\delta d_{\mathrm{A}}(i) = \left|\left\{g_j \mid g_j \in Y, g_i \in X, a_{ij}\, \mathbf{u}_{\mathrm{X}}(i)\, \mathbf{u}_{\mathrm{Y}}(j) > 0, s(X,Y) > \alpha\right\}\right|.$$

We normalize this quantity considering the genes g_j, such that:

- g_j belongs to Y, a set that establishes a crosstalk with X, which contains g_i;
- g_j interacts with g_i.

Formally:

$$d_{\mathrm{A}}(i) = \left|\left\{g_j \mid g_j \in Y, g_i \in X, a_{ij} = 1, C(X,Y) > 0\right\}\right|.$$

The *saturation* of the interaction diversity of g_i is:

$$r_{\mathrm{A}}(i) = \frac{\delta d_{\mathrm{A}}(i)}{d_{\mathrm{A}}(i)}.$$

Supplementary Figures

S1 Fig. Gene weights, gene-gene interactions and crosstalk values of four gene sets pairs that exemplify the four combinations of evidence about the statistical significance of a crosstalk based on the two null models M_{A} and M_{u}. **a-d).** Average weight $|\mathbf{u}|$ (horizontal axis) of all the genes that are involved in the crosstalk between a pair of gene sets X and Y, and number of interactions $|\mathbf{A}|$ (vertical axis) between the genes of X and those of Y, in 1000 degree-preserving permutations of gene labels applied to gene weights (null model M_{u}) or gene-gene interactions (null model M_{A}). **e-h).** Crosstalk values in null models M_{u}(horizontal axis) and M_{A} (vertical axis) for the same gene sets pairs and permutations shown in panels a-d. **a-h)** The analysis was performed as described in the main text section "Alteration of intra-cellular crosstalks in cancer active fibroblasts". **a,e)** (OXIDATIVE_PHOSPHORYLATION, P53_PATHWAY): not significant in any null. **b,f)** (E2F_TARGETS, G2M_CHECKPOINT): significant in M_{A}; **c,f)** (MYC_TARGETS_V1, OXIDATIVE_PHOSPHORYLATION): significant in M_{u}; **d,h)** (ALLOGRAFT_REJECTION, MYC_TARGETS_V1): significant in both nulls.

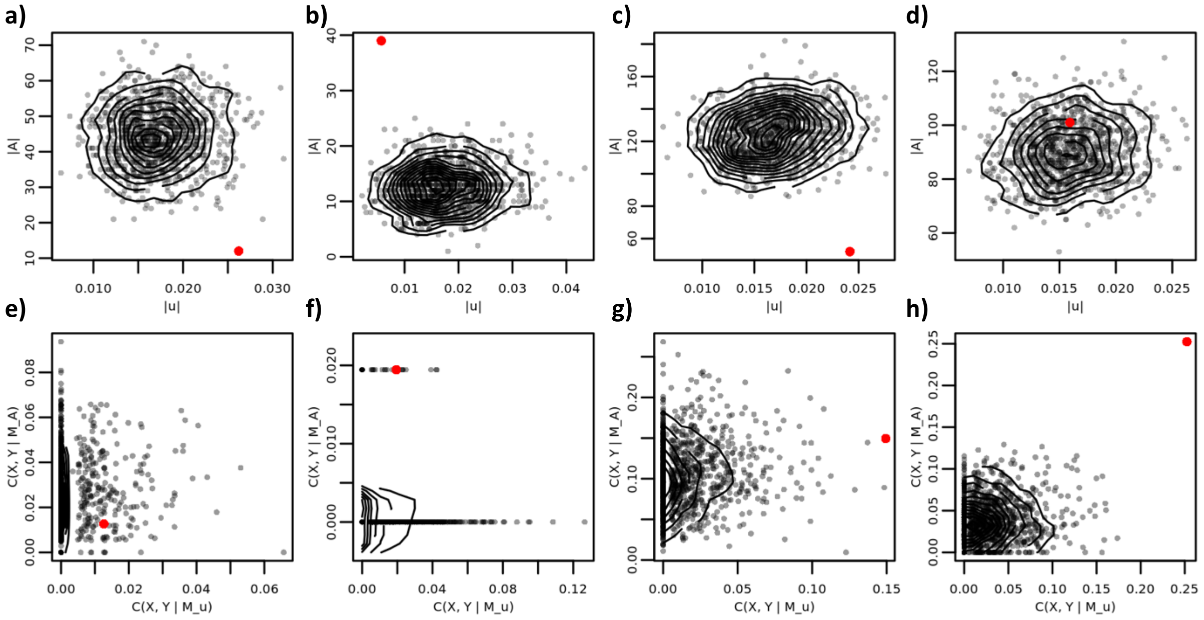

S2 Fig. Frequency distribution of the Spearman correlation values between the two null models $M_{\mathbf{A}}$ and $M_{\mathbf{u}}$.

The correlations shown are between crosstalk values (1000 permutations) in the two null models. Crosstalks were calculated as described in the main text section “Alteration of intra-cellular crosstalks in cancer active fibroblasts”.

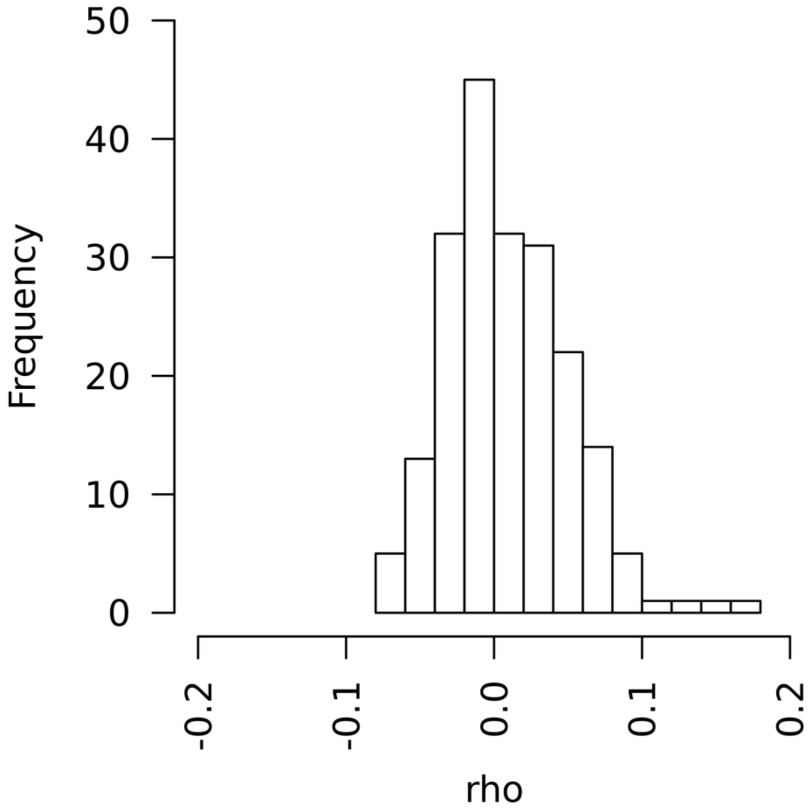

S3 Fig. Comparison of cell clusters and cell type annotation of the 8 TNBC samples used in our proof-of-concept.

On the left, the cell clustering performed by Pal *et al*. (2021) [3] and provided as the Seurat object "SeuratObject_TNBC.rds" [4]. On the right, the cluster annotation reconstructed by us based on what is written in Pal *et al*. (2021) [3] and what is available in the Seurat object "SeuratObject_TNBCSub.rds" [4], as described in the main text.

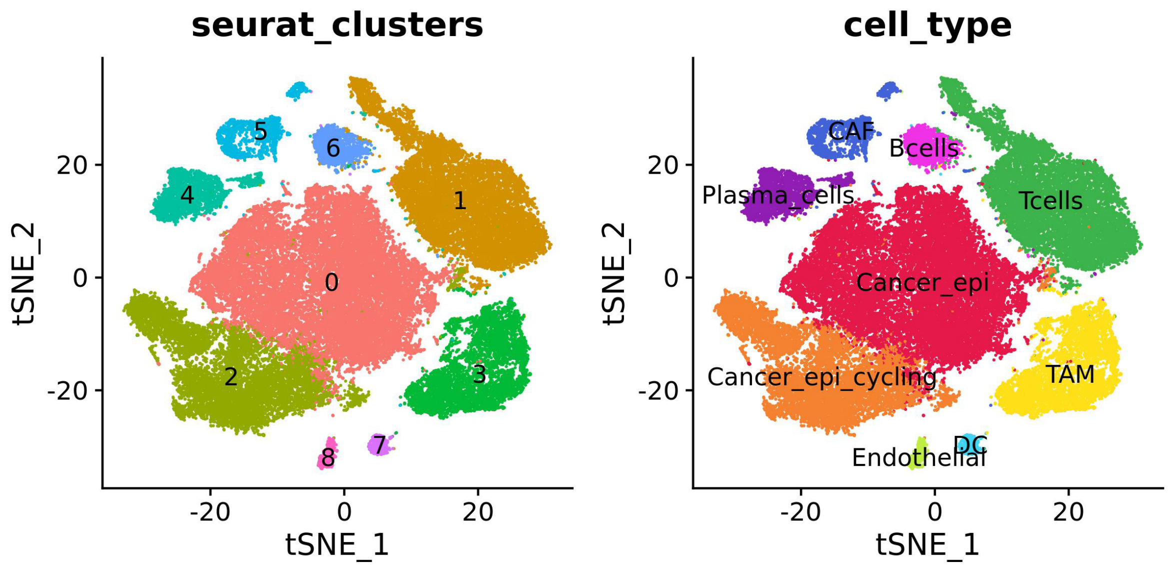

Supplementary Tables

S1 Table. Estimated probabilities of the crosstalk values reported in S1 Fig.

X	Y	ρ_{A}	ρ_{u}	p
OXIDATIVE_PHOSPHORYLATION	P53_PATHWAY	0.799	0.108	0.298
E2F_TARGETS	G2M_CHECKPOINT	0.030	0.228	0.041
MYC_TARGETS_V1	OXIDATIVE_PHOSPHORYLATION	0.132	0.001	0.001
ALLOGRAFT_REJECTION	MYC_TARGETS_V1	0.001	0.001	$\sim 10^{-5}$

S2 Table. Differential expression analysis results.

The table contains the results obtained with "FindAllMarkers()" function form Seurat, together with the cell type annotation and score u used in Ulisse analysis, calculated as described in Method. See file "SupplementaryTables_2-10.xlsx".

S3 Table. Differential expression genes (DEG) mapping data.

The table contains data about the number of genes tested and DEGs mapped to STRING, Omnipath networks and Hallmarks gene sets, for each cell type. See file "SupplementaryTables_2-10.xlsx".

S4 Table. Intracellular crosstalk results in Cancer cells.

The table contains the analysis results obtained from Ulisse, listing: names of the gene sets ("S1_name", "S2_name"), crosstalk score ("c"), sizes of the gene sets ("S1_size", "S2_size") and the subset involved in the crosstalk calculation ("S1_S2_size", "S2_S1_size" = number of genes; "S1", "S2"= gene names; "u1", "u2" = sum of weights u of the genes involved), number of links between the pairs ("dL" = affected; "L" = possible) and their crosstalk saturation ("r_c"), summary score ("s"), and p-values ("pA", "pU" and their combination "p"). The last column ("significance") indicates whether each pair is supported by one ("M_A | M_U < 0.01"), both ("M_A & M_U < 0.01") or no ("M_A & M_U > 0.01") null models. See file "SupplementaryTables_2-10.xlsx".

S5 Table. Over-Representation Analysis (ORA) on cancer cells DEGs results.

The table contains the results of the ORA on Hallmarks gene sets, by listing the number of tested genes ("N"), DEGs ("wb"), non-DEG genes ("bb"), genes in the gene set ("bd") and DEGs in gene set ("wbd"), together with expected value ("exp"), enrichment ("er") and statistical evaluation ("p_val", "p_adj", "q_val"). See file "SupplementaryTables_2-10.xlsx".

S6 Table. Gene classification of cancer intracellular crosstalk.

The table shows the results of gene classification analysis performed on significant crosstalk, as described in Methods. Data listed in the columns are: crosstalk diversity

(“ddX”), saturation of crosstalk diversity (“rX”), gene sets involved (“ddX_S”), gene set measures used for crosstalk diversity normalization (“dX” = number of gene set, “dX_S” = gene set involved in dX), interactor diversity (“ddA”), saturation of interactor diversity (“rA”), genes involved (“ddA_gene”) and genes measures used for interactor diversity normalization (“dA” = number of genes, “dA_gene” = genes involved in dA), initial weight of the gene (“u”). See file “SupplementaryTables_2-10.xlsx”.

S7 Table. Intercellular crosstalk results.

The table contains the results of intracellular crosstalks analysis performed as described in Methods. Column legend as in S4 Table. See file “SupplementaryTables_2-10.xlsx”.

S8 Table. Cancer-CAF intercellular crosstalk details.

This table contains data about the communication between cancer cells and CAF, with the detail of the gene-gene and score (“u12”, calculated as product of gene weights) of their interaction. See file “SupplementaryTables_2-10.xlsx”.

S9 Table. Intercellular crosstalk gene classification.

Here are listed the results of the gene classification analysis performed on significant intercellular communications, as described in Methods. Column legend as in S6 table. The last column (“specific”) indicates if a gene is present among cancer cell (“Cancer_epi”), CAF or both communicating gene sets, or none of them (“other”). See file “SupplementaryTables_2-10.xlsx”.

S10 Table. Integrated crosstalk analysis in cancer cells, involved in communication with CAF.

The table shows the result of integrated crosstalk analysis. Column legend as in S4 table. In addition, “ccc” column indicates which communication is analysed, and “S1_name” represents cancer cells genes involved in the communication with CAFs. The last column (“intracellular”) indicates if the pathway in “S2_name” is involved in significant intracellular crosstalk. See file “SupplementaryTables_2-10.xlsx”.

References

1. Wallis WA. Compounding Probabilities from Independent Significance Tests. Econometrica. 1942;10: 229. doi:10.2307/1905466
2. Fisher RA. Statistical Methods for Research Workers. 1992. pp. 66–70. doi:10.1007/978-1-4612-4380-9_6
3. Pal B, Chen Y, Vaillant F, Capaldo BD, Joyce R, Song X, et al. A single-cell RNA expression atlas of normal, preneoplastic and tumorigenic states in the human breast. EMBO J. 2021;40. doi:10.15252/embj.2020107333
4. Chen Y, Smyth G. Data, R code and output Seurat Objects for single cell RNA-seq analysis of human breast tissues. In: figshare. 2022. doi:10.6084/m9.figshare.17058077.v1
