# Supplementary material for: Analysis of intracellular and intercellular crosstalk from omics data
Source: PLoS One. 2025 Oct 22;20(10):e0334981. doi: 10.1371/journal.pone.0334981 (PMC12543113; doi:10.1371/journal.pone.0334981)
Supplement: Attachment — Submitted filename: rebuttal_letter.pdf [file pone.0334981.s003.pdf]

Dear Editor,

below you will find our point-by-point answers (red) to reviewers' comments (*italic*).

Kind Regards,

Ettore Mosca

****

Reviewer #1: *The manuscript by Chiodi et al. proposes Ulisse, a pipeline of analyses aimed at quantifying molecular cross-talk between pathways and cell types. Furthermore, the authors leverage the defined cross-talk statistics to classify genes according to how they mediate the cross-talk.*

I have found the ideas presented in this paper quite interesting and of wide applicability in network medicine. However, several points should be addressed before publication:

We thank the reviewer for acknowledging the attractiveness and wide applicability of our ideas in network medicine.

1) Have the authors considered a metric of interaction beyond the simple direct interaction or link reported in A? For example, what about leveraging the inverse of the shortest path or a function of a diffusion-based distance? This would allow a more comprehensive assessment of "cross-talk", beyond the presence or absence of a single link.

The reviewer proposes an interesting approach, which we have already explored in a previous work (Mosca et al, 2021 Briefings in Bioinformatics, 10.1093/bib/bbab153), where we quantified the distance between two pathways based on the average network diffusion scores among all their components. Nevertheless, we acknowledged that the simplification of the topological complexity of the two pathways into a single score, although potent, presents a more intricate interpretation, other than introducing biases specific to the mathematical formulation chosen for the score.
In our new work, we aimed at providing an intuitive score that can be easily understood in terms of the molecular alterations and interactions involved in the crosstalk. At the moment, we discuss the possible application of network diffusion over the input values, to amplify the scores of genes in network proximity.

2) I suggest the authors give straightforward examples of a set of genes of interest, of an array of gene sets, of a possible adjacency matrix, and of potential weights connecting the selected genes of interest and the gene sets when they first define the cross-talk score. I am aware the authors have a proof-of-concept and a supplementary material. Yet, the net outcome is that the key information is diluted over several pages without being presented cohesively.

Thanks for the suggestion. We thoroughly updated the manuscript, and one of the aims was at improving its clarity. Among the new content, there is a Table that illustrates the top 5 crosstalks.

3) *The null model behind the empirical p-value deserves more work and/or a better explanation.*

- *It is unclear how the original score, calculated with weights, can be compared with randomized calculations that set weights to 1. Can you clarify this point?*
- *I understand the creation of random gene set pairs with sizes equal to the original gene sets. However, the sum in the original equation does not run over all genes in the gene sets, but over genes of interest in the gene sets. How do you take this into account?*
- *Considering the properties of A, have you tested degree-matching randomizations (through log-binning)? This would be implemented by sampling sets of the original size from the network generated by A, and by selecting nodes within the same degree bins populated by the original nodes.*

This is a key aspect that we substantially improved. We introduced two null models, to assess the association of the observed score with interactions and gene weights. The use of multiple null models allows us to systematically sample alternative network configurations and test the effect of a single feature over the outcome. Interestingly, probing this outcome from multiple angles suggests possible links between network features and biological meaning, that is: is the cross-talk score due to interactions or magnitude of gene weights? The null models are based on permutations that preserve gene set size, degree sequence, and the association between gene weight and gene degree (within the same bin over the degree sequence).

4) The authors should consider comparing their measure of functional diversity, interaction diversity, and functional relevance with centrality metrics on A, such as degree, betweenness centrality, eigenvector centrality, etc. This would complement their analysis, highlighting the additional information they are able to capture.

We rewrote the article to clarify the use of gene-level scores. We consider the cross-talk diversity and interaction diversity in relation to their saturation (to enable comparison between different absolute values). We discuss the fundamental difference between these quantities and the purely topological features mentioned by the reviewer.

5) Quantifying the cross-talk between pathways could be used as a methodology to assess the dependency in multiple testing. I think this would be a nice addition to the discussion.

This is an interesting suggestion. In the new version we compared crosstalk analysis with pathway analysis and found that the two analyses provide a complementary view of the occurrence of molecular alterations over pathways. The outcomes of the proof-of-concept study support the independence of the two analyses. Therefore, a more comprehensive study of the topic is required to assess whether the reviewer's intuition holds.

****

Reviewer #2: *In this manuscript by Chiodi and colleagues, a general framework to quantify cellular cross-talk is considered with focus on both pathway cross-talks and intercellular cross-talks. The premise for the work is on the use of ever-growing gene expression based measurements to infer interactions among pathways and genes, their functional relevance in the context of disease, and possibly identify the critical gene nodes that could be targeted for pharmacological benefits, and phenotype switching. The manuscript describes a general framework, applies the framework to pathway analysis, explores the disease relevance in breast cancer subtypes, and compares the performance of the approach to other existing methods. The rationale to identify critical players from omics data that can be functionally perturbed for biological understanding of the disease or cellular function is described as motivation behind the study.*

While I appreciate the general enthusiasm towards inferring the cross-talk in omics data, I am not positive about what added information Ulise provides on top of the current existing methods. The performance of Ulisse seems at best similar to the current methods IcellNet etc. Furthermore, authors indicate the method can shed light on additional layers of complexity currently missed by existing methods, I am not sure if the key players identified as it was applied to breast cancer subtypes, indicating any new, surprising, or unintuitive conclusion. Some of the analysis to identify cell-cell communication for microenvironment is what we already know of the interactions in that system, so what additional information the method provides is unclear. I also find the implementation of Ulisse with different metrics (FDR, Score, DEG score) resulting in different performance rather puzzling. I understand the choice of metrics matter, but authors don't have any recommendation on the metrics for further analysis that results in robust results. Various metrics of Ulisse show poor correlations compared to Ulisse score and ICN, does this mean, the Ulisse score should be considered as a superior way of ranking the gene lists?

Overall, I am not sure if the method provides any advancement to the current state of cell-cell communication frameworks. However, this work, at its present form, can lay a strong foundation for inferring joint intra-, and intercellular cross-talks that authors rightly pointed in the Discussion.

We substantially revised our work to clarify what our method adds on top of the current existing methods. The newly developed proof-of-concept effectively elucidates the nature of information that the method delivers. Among the novelties that are worth noting, we implemented the joint analysis of intra- and inter-cellular crosstalk.
